# Brian Edmond McConnell

**Published:** 1964-07

**Authors:** 


### *Obituary*

### BRIAN EDMOND MCCONNELL M.D., CH.B., B.A.O., M.R.C.P.

It is with deep regret that we record the death on 29th May 1964, at the early age of 48, of Dr. Brian McConnell, one of Bristol's best known and respected practitioners. He will be sadly missed by his very wide circle of friends and acquaintances, not only in the medical profession but in the broader social world beyond it.

A personal friendship which was to last for over twenty years began during the war in a disused Cornish sea-side boarding house where, as fellow officers in the R.A.M.C. we shared common premises. He was then a junior specialist in ophthalmology, having qualified at Belfast in 1939. He enjoyed his work but it was obvious that his wide interests could never be confined within a narrow speciality and it was no surprise to find after demobilization that he had taken up the study of pathology. He worked for several years in the pathology departments of Bristol Royal Infirmary and the University of Bristol and during this time he obtained both the M.R.C.P. and, by examination, the M.D. of his own University of Belfast. Too interested in people to find permanent satisfaction in the relatively inanimate sphere of laboratory work, he entered general practice and here, one felt, he was at last able to give full expression to his broad, down-to-earth, approach to the practice of medicine in its widest sense. Impeccably tidy in appearance, thought and action he brought to his work an eminently practical mind, backed by a deep and fundamental knowledge of his subject. He won deep respect not only from his patients but all his colleagues throughout the profession.

While he devoted himself wholeheartedly to his patients and their problems he never lost touch with "academic" medicine and was a most assiduous attender of clinical and medical meetings of all kinds. He was a member of the British Medical Association and played a full part in local medical affairs. A prominent member of the Bristol Medico-Chirurgical Society, he bore with distinction for many years the office of its Honorary Secretary.

Outside the realm of medicine his activities seemed limitless. There was hardly a subject in which he was not interested and one might say that his principal recreation was conversation. One could never meet him without being treated to the latest of an apparently inexhaustible fund of truly amusing stories. Another hobby was golf and he was also a keen freemason, being at the time of his death immediate Post Master of St. Vincent Lodge.

The affection and esteem in which he was held by his patients and colleagues was reflected in the congregation numbered in hundreds which paid their respects at the funeral service. We extend our deepest sympathy to his wife, and their three children.

